# *Lactobacillus rhamnosus* GG in the Primary Prevention of Eczema in Children: A Systematic Review and Meta-Analysis

**DOI:** 10.3390/nu10091319

**Published:** 2018-09-18

**Authors:** Hania Szajewska, Andrea Horvath

**Affiliations:** Department of Paediatrics, The Medical University of Warsaw, Żwirki i Wigury 63A, 02-091 Warsaw, Poland; andrea.hania@gmail.com

**Keywords:** probiotics, allergy, infants, pediatrics

## Abstract

Current guidelines recommend the use of probiotics to reduce the risk of eczema. It remains unclear which strain(s) to use. We systematically evaluated data on the efficacy of *Lactobacillus rhamnosus* GG (LGG) supplementation prenatally and/or postnatally for the primary prevention of eczema. The Cochrane Library, MEDLINE, and EMBASE databases were searched up to August 2018, with no language restrictions, for systematic reviews of randomized controlled trials (RCTs) and RCTs published afterwards. The primary outcome was eczema. For dichotomous outcomes, we calculated the risk ratio (RR) and 95% confidence interval (CI). A random-effects model was used to pool data. Heterogeneity was explored using the I^2^ statistics. The GRADE criteria were used to assess the overall quality of evidence supporting the primary outcome. Seven publications reporting 5 RCTs (889 participants) were included. High to moderate certainty in the body of evidence suggests that LGG supplementation (regardless of the timing of administration) did not reduce the risk of eczema. There was also no consistent effect on other allergic outcomes. This meta-analysis shows that LGG was ineffective in reducing eczema. It does not support the general recommendation to use probiotics for preventing eczema, unless specific strains would be indicated.

## 1. Introduction

Allergic diseases are a major public health concern in many countries [1]. Among other factors, disturbances in gut microbiota composition and/or activity (dysbiosis) may contribute to the pathogenesis of allergic diseases [2]. If so, gut microbiota may be a target for improving outcomes in subjects affected or at risk for allergic diseases. To date, modification of gut microbiota via the provision of probiotics and/or prebiotics is the most extensively studied strategy.

Probiotics are live microorganisms that, when administered in adequate amounts, confer a health benefit on the host [3]. The mechanisms of action of probiotics remain unclear. However, protolerogenic action of probiotics in the gastrointestinal tract has been suggested [4]. First, probiotics compete with pathogenic organisms for nutrients and binding sites on the intestinal epithelium. Second, they may secrete bacteriocins and induce intestinal epithelium to secrete defensins, natural antimicrobial peptides. Third, through fermenting fibers, probiotics stimulate the production of metabolites such as short-chain fatty acids, the majority of which are acetate, propionate, and butyrate. Short-chain fatty acids activate G protein-coupled receptors that stimulate colonic dendritic cells and macrophages to secrete interleukin-10 (IL-10) and promote development of regulatory T lymphocytes (Tregs) in the mesenteric lymph nodes. Tregs are a source of tolerogenic cytokines such as IL-10 and transforming growth factor-beta (TGF-beta) that inhibit allergic and inflammatory responses [4].

A 2014 guideline by the World Allergy Organization (WAO) [5] did not recommend use of probiotics for reducing the risk of allergy in children. However, the WAO considered that there is a likely net benefit from using probiotics for preventing eczema. Specifically, the WAO suggests: “*(a) using probiotics in pregnant women at high risk for having an allergic child; (b) using probiotics in women who breastfeed infants at high risk of developing allergy; and (c) using probiotics in infants at high risk of developing allergy”*. All recommendations were conditional and supported by a very low quality of evidence. Since the beginning, these guidelines raised a debate [6], mainly because of the lack of answers to practical questions such as: Which probiotic(s) should be used to reduce the risk of eczema? When should one start the administration of probiotics with proven efficacy? When should one stop? What is the dose of an effective probiotic [7]? Our aim was to systematically evaluate evidence on the efficacy of *Lactobacillus rhamnosus* GG (LGG) supplementation during the prenatal and/or postnatal period for the reducing the risk of eczema. The effect on other allergic diseases was also evaluated. LGG is widely available and commonly used in the pediatric population. It is also the first probiotic for which a reduction in the risk of atopic eczema has been documented [8].

## 2. Materials and Methods

The methodology was similar to one followed in our earlier systematic review on allergy prevention [9]. The guidelines from the Cochrane Collaboration for undertaking and reporting the results of this systematic review and meta-analysis were followed [10].

### 2.1. Criteria for Considering Studies for This Review

#### 2.1.1. Type of Studies

Randomized controlled trials (RCTs) were considered for inclusion.

#### 2.1.2. Type of Participants

Participants had to be healthy (1) pregnant women at high risk for having an allergic child; (2) breastfeeding mothers of infants at high risk of developing allergy; or (3) healthy term infants at high risk of developing allergy.

#### 2.1.3. Type of Interventions

We included trials that compared use of the LGG compared with placebo or no intervention. If other experimental arms were available, they were not considered.

#### 2.1.4. Type of Outcomes

Our primary outcome was eczema. However, we also focused on the other allergic manifestations such as wheezing/asthma, allergic rhinitis, food allergy (all as defined by the authors of the original publications), and adverse events. We report outcomes at time intervals reported by the authors of the original publications (or as close as possible).

### 2.2. Search Methods for Identification of Studies

First, we identified RCTs via reviewing previously completed systematic reviews [11,12]. As there was no discrepancy between two recent reviews with regard to LGG studies, we only searched for RCTs published subsequently to these reviews. The Cochrane Central Register of Controlled Trials (CENTRAL, the Cochrane Library), MEDLINE, and EMBASE databases were searched for relevant studies from December 2014 (end date of last search in the first systematic review [9]) to August 2018. There were no language restrictions. The search was carried out independently by two reviewers. In brief, the following search terms were used: (“infant, newborn”(MeSH Terms) OR (“infant”(All Fields) AND “newborn”(All Fields)) OR “newborn infant”(All Fields) OR “neonat*”(All Fields) OR “infant”(MeSH Terms) OR “infant”(All Fields) OR “pediatric”(All Fields) OR “paediatric”(All Fields)) AND (“hypersensitivity”(MeSH Terms) OR “hypersensitivity”(All Fields) OR “allergy”(MeSH Terms) OR “allergy”(All Fields) OR “allergy and immunology”(All Fields) OR “food allergy”(All Fields) OR “milk allergy”(All Fields) OR “eczema”(MeSH Terms) OR “eczema”(All Fields) OR “wheezing”(All Fields)) OR “asthma”(MeSH Terms) OR “asthma”(All Fields)) AND (*Lactobacillus* (All Fields)).

Additionally, we searched reference lists from identified studies and key review articles. Experts in the field were contacted for additional references.

#### 2.2.1. Selection of Studies

Two reviewers initially screened the title, abstract, and keywords of every record identified with the search strategy, and they retrieved the full texts of potentially relevant trials and of records for which the relevance was unclear. The same reviewers independently applied the inclusion criteria to each potentially relevant trial to determine its eligibility. If differences in opinion existed, they were resolved by discussion until a consensus was reached.

#### 2.2.2. Data Extraction and Management

Data extraction was performed using standard data-extraction forms. In addition to data such as methods, participants, interventions, and outcomes (including the definitions of the primary outcome of interest used in the study), we collected information about sample size calculation and the funding of each study. One reviewer extracted the data from the included studies, and the second author checked the extracted data. Discrepancies between the reviewers were resolved by discussion until a consensus was reached. Participants, interventions, comparisons, and outcomes were taken into consideration to determine whether they were similar enough to allow pooling of data.

#### 2.2.3. Assessment of Risk of Bias in Included Studies

The Cochrane Collaboration’s tool for assessing risk of bias was used. The risk of bias parameters included the type of randomization method (selection bias), allocation concealment (selection bias), blinding of participants and personnel (performance bias), blinding of outcome assessment (detection bias), and incomplete outcome data (attrition bias). Additionally, selective reporting (reporting bias) and other types of bias were considered. If an item could not be evaluated due to missing information, it was rated as having an unclear risk of bias [13].

#### 2.2.4. Dealing with Missing Data

We assessed pooled data using intention-to-treat analysis, i.e., an analysis in which data are analyzed for every participant for whom the outcome was obtained (also known as available case analysis), rather than intention-to-treat analysis with imputation [14].

#### 2.2.5. Assessment of Heterogeneity

Heterogeneity was quantified by *χ*^2^ and *I*^2^. A value for *I*^2^ of 0% indicates no observed heterogeneity, and larger values show increasing heterogeneity. All analyses were based on the random effects model.

#### 2.2.6. Assessment of Reporting Biases

To test for publication bias, a test for asymmetry of the funnel plot, as proposed by Egger et al. [15], was planned; however, sufficient (≥10) eligible trials were not available for any given outcome.

#### 2.2.7. Data Synthesis

The data were entered into Review Manager (RevMan), Computer program, Version 5.3. (The Nordic Cochrane Centre, The Cochrane Collaboration, Copenhagen, Denmark) for analysis. The results for individual studies and pooled statistics are reported as the risk ratio (RR) between the experimental and control groups with 95% confidence intervals (95% CI). RR is significant when the 95% CI does not include 1.0.

#### 2.2.8. Subgroup Analysis

Subgroup analysis was based on the timing of the intervention (prenatally only, prenatally and postnatally, postnatally only).

#### 2.2.9. Quality of Evidence

For assessing the quality of evidence (also known as certainty in the evidence or confidence in the effect estimates) for the primary outcome, we chose to use the GRADE methodology and GradePro software, GRADEpro GDT: GRADEpro Guideline Development Tool (Software). (McMaster University, Evidence Prime, Inc., Hamilton, ON, Canada). This software is available from gradepro.org. The GRADE system offers 4 categories of the quality of the evidence (i.e., high, moderate, low, and very low).

## 3. Results

### 3.1. Description of Studies

We identified seven publications [8,16,17,18,19,20,21] reporting on 5 RCTs (Table S1), which involved 889 participants at enrollment (443 in the LGG group and 446 in the control group). Compared to previously published systematic reviews that identified 8 publications on LGG, we excluded the study by Rautava et al. [22], as it reported data on a subset of the population from the study by Kalliomaki et al. [8]. For a flow diagram documenting the identification process for eligible trials, see Figure S1.

All studies were carried out in high-income countries such as Australia (one), Finland (one), Germany (one), Taiwan (one), and the US (one). One trial reported data on the administration of LGG during late pregnancy only [17]. Three trials [8,20,21] reported data on the administration of LGG during pregnancy and to infants (duration of intervention prior to delivery ranged from 14–18 weeks to 2–4 weeks, and in all trials was for 6 months after delivery). One RCT [18] reported data on the administration of LGG to infants only (the duration of intervention was 6 months). The risk of allergy was assessed in the included trials by a family history (the presence of allergy in at least one parent and/or sibling) and/or other markers.

The daily doses of LGG ranged from 1.0 × 10^9^ colony forming units (CFU) to 1.8 × 10^10^ CFU. The sample size ranged from 105 to 250. Four RCTs were placebo-controlled. In one trial, inulin was administered in both study groups, thus, any effect, if it exists, may be attributable to LGG. All included RCTs evaluated eczema. However, various definitions were used. Atopic eczema IgE-associated was reported separately in one trial only [16]. Other allergic manifestations were reported inconsistently. In case of wheezing and asthma, the definitions were often overlapping, hence, our decision to present these data jointly.

#### Risk of Bias in Included Studies

**Figure 1:**
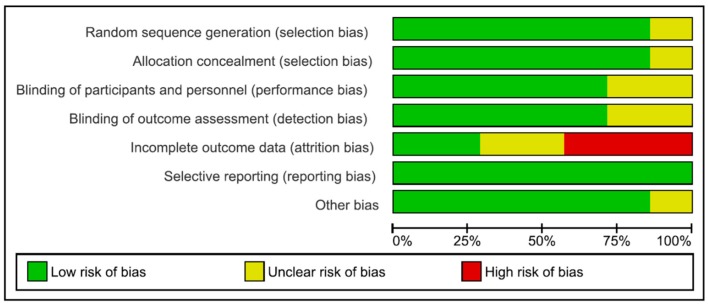
Risk of bias graph.

**Figure 2:**
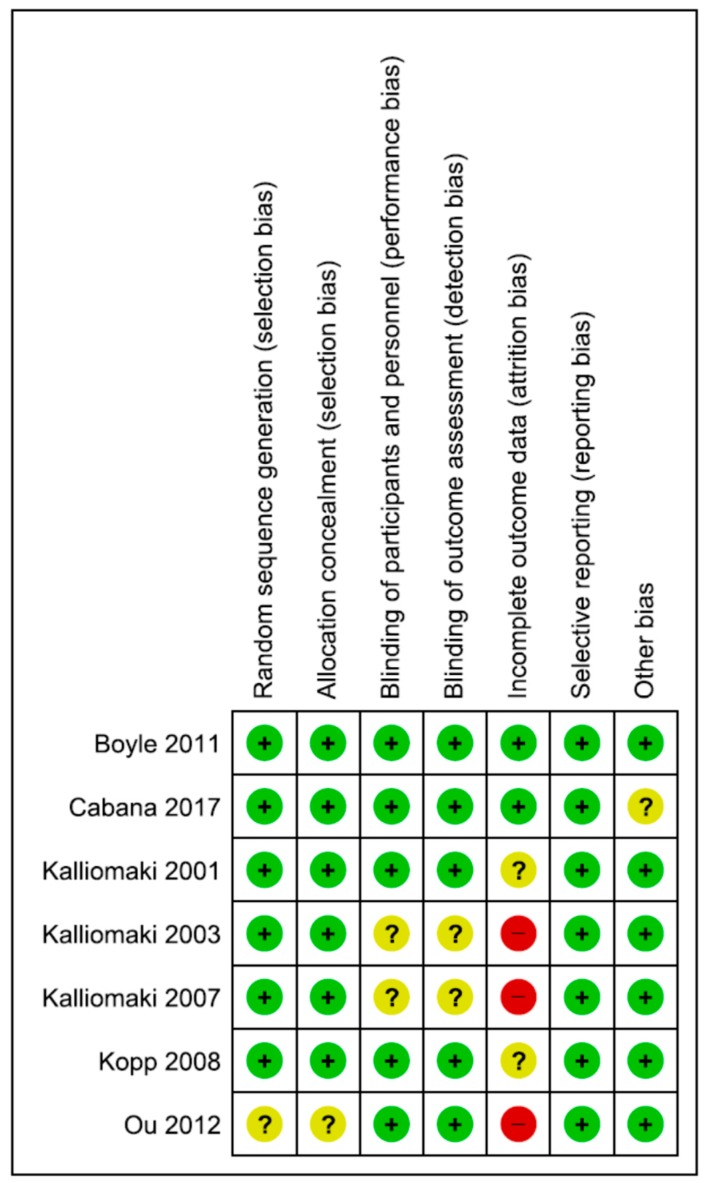
Risk of bias summary.

The included studies are described with respect to their risk of bias across the included RCTs in Figure 1 and Figure 2. Only one trial [17] had a low risk of bias. The remaining trials had some methodological limitations. Sample size calculations were performed in all 5 trials. Funding was reported in 4 trials.

The GRADE assessment for the primary outcome related to use of LGG is presented in Table S2. Using the GRADE methodology, the overall quality of evidence was rated as high to moderate.

### 3.2. Effects of Interventions

#### 3.2.1. Eczema

**Figure 3:**
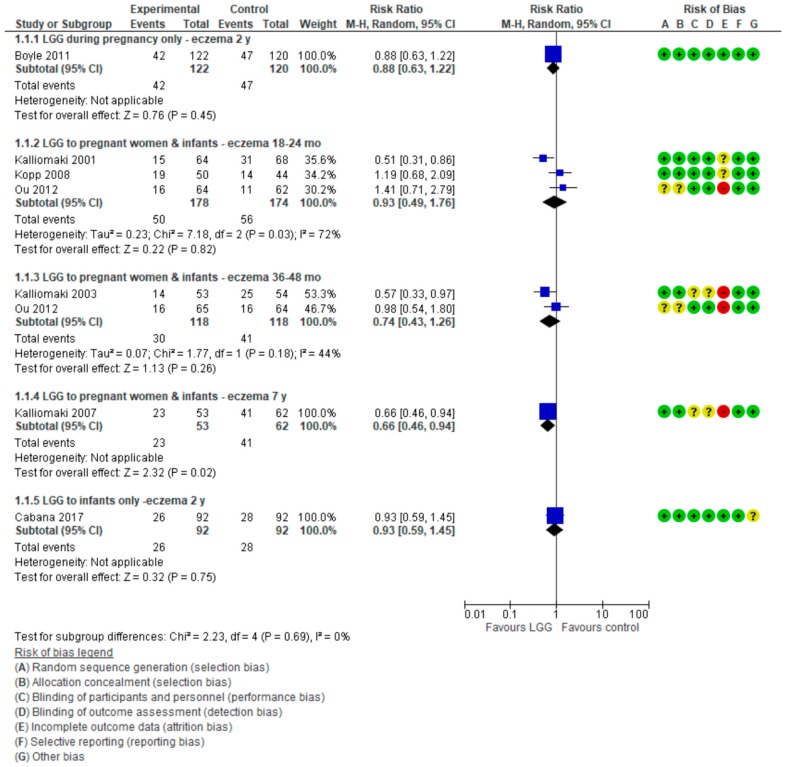
Primary outcome: Effect of LGG supplementation on eczema (data presented based on the timing of LGG administration and the timing of assessment).

For data on eczema, see Figure 3.

##### LGG Administration during Late Pregnancy

Only 1 RCT (*n* = 242) [17] reported the effect of use of LGG administration during pregnancy on the cumulative incidence of eczema and found a similar risk in both study groups (RR 0.88 (95% CI: 0.63, 1.22)).

##### LGG Administration during Pregnancy and after Delivery

Three RCTs [8,20,21] reported the effect of LGG administration during pregnancy and to infants on the cumulative incidence of eczema. The pooled results of data up to 2 years of age showed no reduction in the risk of eczema (3 RCTs, *n* = 352, RR. 0.93 (0.49, 1.76)). Significant heterogeneity was found (*I*² = 72%). There also was no difference between groups in the risk of eczema in children up to 3–4 years (2 RCTs, *n* = 236, RR 0.74 (0.43, 1.26); *I*² = 44%). Only one RCT (*n* = 115) reported a significant reduction in the risk of eczema in children up to 7 years in favor of the LGG group (RR 0.66 (0.46, 0.94)) [19]. However, only 72% (115 of 159) of participants were followed up at 7 years.

##### LGG Administration to Infants Only

One 1 RCT [18] in which LGG was administered to infants only reported no significant difference between groups in the risk of eczema up to 2 years of age (RR 0.93 (0.59, 1.45)).

The pooled results of 5 RCTs [8,17,18,20,21] (*n* = 778) showed no difference between the LGG-supplemented and control groups in the risk of eczema, regardless of the timing of LGG administration, up to 2 years (RR 0.90 (0.67, 1.21), *I*² = 45%).

#### 3.2.2. Wheezing/Asthma

**Figure 4:**
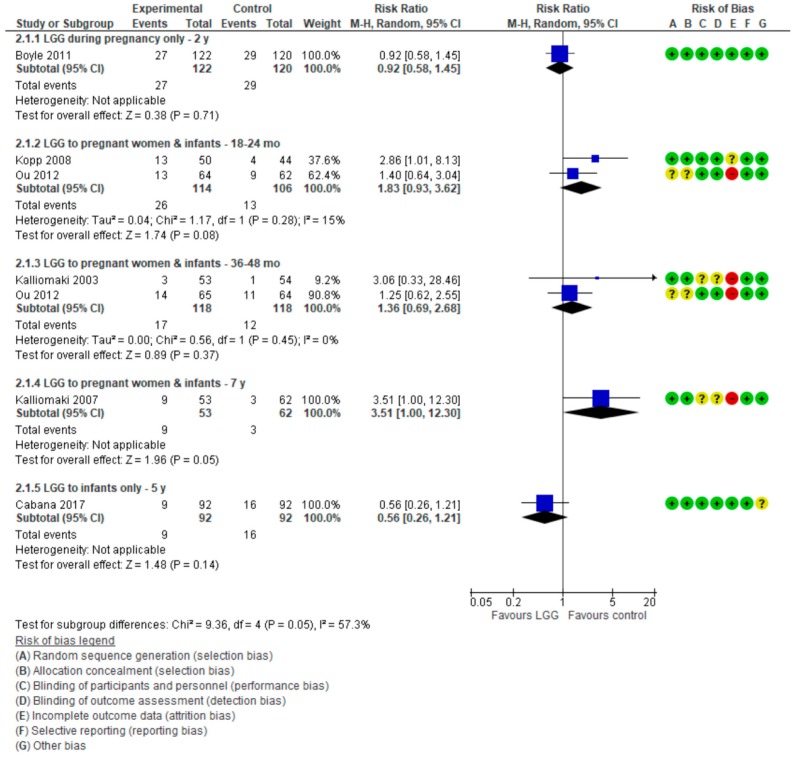
Secondary outcome: Effect of LGG supplementation on wheezing/asthma (data presented based on the timing of LGG administration and the timing of assessment).

For data on wheezing/asthma, see Figure 4.

##### LGG Administration during Late Pregnancy

One RCT [17] in which LGG was administered only during late pregnancy reported no effect of LGG administration on the risk of wheezing in children up to 2 years of age (RR 0.92 (0.58, 1.45)).

##### LGG Administration during Pregnancy and after Delivery

Four publications reporting on 3 RCTs [16,19,20,21] reported the effect of LGG administration during pregnancy and to infants on the cumulative incidence of wheezing/asthma at various time intervals. At 7 years, an increase in the risk of asthma in the LGG group compared with placebo group was observed; however, this difference was of a borderline significance (RR 3.51 (1.00, 12.30)). Furthermore, in addition to the previously stated high attrition rate in this trial (28%), the very wide confidence intervals call for caution when interpreting these findings. For other time intervals, there were no significant differences between the study groups.

##### LGG Administration to Infants Only

One RCT [18] in which LGG was administered only to infants reported no significant difference between groups in the risk of asthma at 5 years (RR 0.56 (0.26, 1.21)).

#### 3.2.3. Allergic Rhinitis/Sneezing

**Figure 5:**
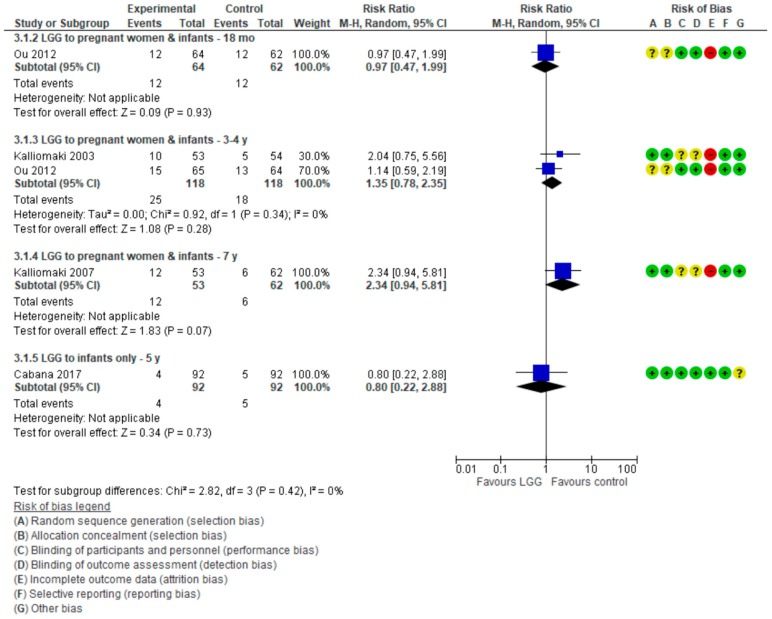
Secondary outcome: Effect of LGG supplementation on allergic rhinitis (data presented based on the timing of LGG administration and the timing of assessment).

For data on allergic rhinitis/sneezing, see Figure 5.

##### LGG Administration during Pregnancy and after Delivery

Three publications [16,19,21] in which LGG was administered during pregnancy and to infants reported data on allergic rhinitis at various time intervals; no significant differences between the study groups were found.

##### LGG Administration to Infants Only

One RCT [18] in which LGG was administered only to infants reported no significant difference between groups in the risk of allergic rhinitis at 5 years (RR 0.80 (0.22, 2.88)).

### 3.3. Food Allergy

None of the trials reported data on food allergy.

### 3.4. Adverse Events

Only 2 RCTs reported data on adverse events. [17] found a non-significant reduction in the risk of adverse event in the LGG group compared with the placebo group (RR 0.66 (0.4, 1.1)). [20] found no difference between the groups (data were not shown).

## 4. Discussion

### 4.1. Summary of Findings

This meta-analysis adds to two recently published meta-analyses [9,10] by focusing on a single, specific probiotic strain. We found no consistent effect of the administration of LGG for reducing the risk of eczema up to 4 years of age, regardless of the timing of LGG administration (during pregnancy and/or during breastfeeding or to infants only). One trial reported a reduced risk of eczema at 7 years of age in the LGG group. However, the 7-year follow-up was completed by only a subset of participants initially randomized into the study (115 of 159, i.e., 72%); hence, this finding had to interpreted with caution. There was no effect of LGG administration on reducing the risk of wheezing/asthma, with the exception of one trial that reported an increased risk of asthma at 7 years in the LGG group compared with the placebo group. However, the high attrition rate in this trial (as mentioned earlier), the borderline statistical significance, and wide confidence intervals around the estimate call for caution in interpreting these findings. There was no effect of LGG administration on allergic rhinitis. Adverse events were similar in both study groups.

### 4.2. Comparison with Other Reviews

Two previously published meta-analyses pooled data on different probiotics [9,10]. A 2015 systematic review (search date: December 2014) by Cuello-Garcia et al. [9] identified 29 publications in which 12 various probiotics, single or in combinations, were used. The authors concluded that there are significant benefits of probiotic supplementation in reducing the risk of eczema when used by women during the last trimester of pregnancy (RR 0.71, 95% CI 0.60 to 0.84), when used by breastfeeding mothers (RR 0.57, 95% CI 0.47 to 0.69), or when given to infants (RR 0.80, 95% CI 0.68 to 0.94). Based on this systematic review, the WAO guidelines were published [5].

A 2018 systematic review (search date: December 2015) concluded that, overall, probiotic supplementation during pregnancy and breastfeeding reduced the risk of both eczema (19 studies; RR 0.78; 95% CI 0.68 to 0.90; substantial heterogeneity was found (*I*^2^ = 61%)) and “atopic” eczema (11 studies; RR 0.78; 95% CI 0.65 to 0.92; no heterogeneity was found (*I*^2^ = 0%) at age ≤ 4 years. However, there was no reduction in the risk of eczema in children aged 5 to 14 years. Subgroup analysis for eczema showed a significant difference between studies that supplemented mothers during the postnatal period (9 interventions; RR 0.64; 95% CI 0.51 to 0.80) and studies that just supplemented infants during the postnatal period (11 interventions; RR 0.93; 95% CI 0.81 to 1.06) [10]. For both meta-analyses, the risk is that pooling data from different genera, species, strains, and doses of probiotics obtained in different settings and/or populations, presumably with variations in their native intestinal microbiota, may result in misleading conclusions. The results could be erroneously extrapolated to other probiotics, including those that have not been adequately studied.

### 4.3. Strengths and Limitations

Our meta-analysis focused exclusively on one type of a clearly defined, single-organism, probiotic microorganism, specifically LGG. Thus, it provides an answer to the question as to whether current evidence should change practice. Nevertheless, several limitations must be emphasized. First, our search depended on the studies identified via reviewing previously completed systematic reviews. However, as the results were consistent, we decided to rely on these searches. Available data were too limited to allow an examination as to whether the timing of probiotic administration matters (i.e., during pregnancy only, or during pregnancy and to infants, or to infants only), even if each timing was assessed separately. All trials were conducted in high-income countries, thus, the generalizability of these findings to less privileged settings remains unclear. Overall, the quality of studies was sound. Still, some methodological issues should be considered when interpreting the results. For example, the high attrition bias in the trial by Kalliomaki et al. [17] is one methodological limitation. However, this was unavoidable in a trial with a 7-year follow-up. Included trials used different definitions of eczema, and atopic eczema was assessed separately in only one trial [15] As the definitions of wheezing and asthma were often overlapping, these data were presented jointly. However, not all cases of early life wheezing will progress into asthma later on. Most children will eventually grow out of the symptoms and will never develop asthma [23]. Regardless, as some trials have indicated an increased risk of wheezing/asthma, more data are needed to evaluate this potentially harmful effect of using probiotics. This is important as a 2018 systematic review found that of nearly 400 RCT interventions aimed at modifying microbiota, only 6% adequately reported harms [24]. Consequently, the safety and potential harms of using probiotics, particularly very early in life and for a prolonged period, remains questionable. Trials included in our review did not report differences in adverse effects. However, only limited data were available.

## 5. Conclusions

This meta-analysis does not support the use of LGG for reducing the risk of eczema. Our findings indicate that current guidelines on the use of probiotics for preventing eczema in infants at high risk for this allergic disease should be revised and be more specific with regard to which strain(s) to use.

## Supplementary Materials

The following are available online at http://www.mdpi.com/2072-6643/10/9/1319/s1, Figure S1: Identification process for eligible trials, Table S1: Characteristics of the included studies, Table S2: GRADE evidence profile summarizing the effect of *Lactobacillus* GG supplementation vs. placebo or no intervention on eczema.
