# Supplementary material for: Lactobacillus rhamnosus GG in the Primary Prevention of Eczema in Children: A Systematic Review and Meta-Analysis
Source: Nutrients. 2018 Sep 18;10(9):1319. doi: 10.3390/nu10091319 (PMC6163317; doi:10.3390/nu10091319)
Supplement: Supplementary file 1 [file nutrients-10-01319-s001.pdf]

Figure S1.

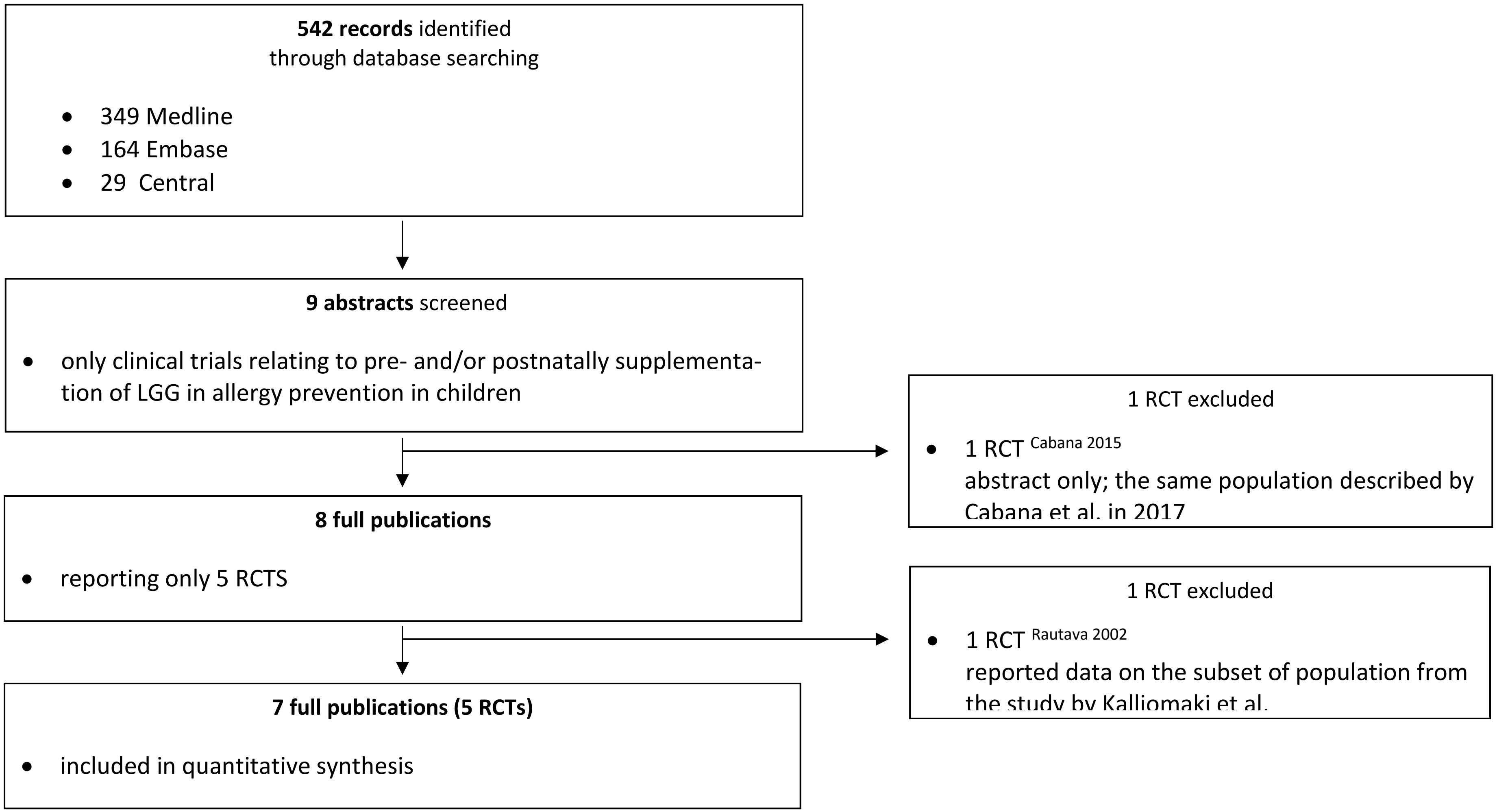
542 records identified
through database searching
349 Medline
164 Embase
29 Central
9 abstracts screened
only clinical trials relating to pre- and/or postnatally supplementation of LGG in allergy prevention in children
8 full publications
reporting only 5 RCTS
7 full publications (5 RCTs)
included in quantitative synthesis
1 RCT excluded
1 RCT [Cabana 2015]
abstract only; the same population described by Cabana et al. in 2017
1 RCT excluded
1 RCT [Rautava 2002]
reported data on the subset of population from the study by Kalliomaki et al.

Table S1

Study	Country	Population [risk of allergy]	Intervention (dose)	Comparison (placebo)	Duration of intervention	Follow up	Definition of allergic manifestations	Outcomes *primary **secondary	Sample size	Funding
**PRENATALLY (only pregnant women supplemented)**										
**Boyle 2011**	Australia	Pregnant women [women, their partner or a previous child with a doctor-diagnosed allergy diseases (eg. asthma, eczema, FA, AR)]	1.8 x 10^{10} CFU (n=125)	Maltodextrin (n=125)	from 36 ws gestation until delivery	12 mo	*Eczema* – a history of itchy skin, scratching or rubbing + at least 3 of the following: family history of atopic disease; history of generally dry skin; history of skin rash affecting the flexures, cheeks or outer surfaces of the limbs; onset of rash < 2 ys of life; visible dermatitis at any study visit affecting the flexures, cheeks or outer surfaces of the limbs<br><br>*Sensitization* – a SPT wheal diameter ≥3 mm greater than the negative control to any single allergen tested<br><br>*Various immunological parameters* – CBMC cytokine secretion, Tregs and DCs	*cumulative incidence of eczema;<br><br>**allergic sensitization; IgE-associated eczema; eczema severity; gastrointestinal and respiratory symptoms	Yes	Yes[1]
**POSTNATALLY (only infants supplemented)**										

**Cabana 2017**	USA	Newborns [participants with at least 1 biological parent who reported a history of asthma]	1.0 x 10^9 CFU + inulin 225 mg (n=92)	Inulin 325 mg (n=92)	for the first 6 mo of life (added to human milk, pHF, water)	5 y	*Eczema* – repeated parental reports of the diagnosis of eczema by a clinician on 2 occasions or the presence of eczema on physical examination on the basis of standard criteria<br><br>*Asthma and AR* - repeated parental reports of the diagnosis by a clinician on 2 occasions	*the incidence of eczema within 2 y of birth<br><br>**the incidences of asthma and AR within 5 y of birth	Yes	Yes[2]
**PRE- (pregnant women) AND POSTNATALLY (breastfeeding mothers and/or newborns)**										
**Kalliomaki 2001**	Finland	pregnant women and their infants [one or more family members (mother, father, or older sibling) with atopic eczema, AR, or asthma]	1 x 10^{10} CFU (n=77)	Microcrystalline cellulose (n=82)	2 to 4 wk before delivery + 6 mo postnatally<br><br>(BF women or infants – added to water)	2 y	*Eczema* – chronic recurring atopic eczema (pruritis, facial or extensor involvement, or both, and chronic relapsing course)<br><br>*AR* – 2 or more of chronic, recurring symptoms: nasal discharge, blockage, sneezing, and itching<br><br>*Asthma* – a chronic or recurrent cough, wheeze or shortness of breath, or both, if other diagnoses were excluded and trial antiasthma treatment was effective	* the frequency of atopic eczema at 2 y of life<br><br>** the frequency of asthma and AR at 2 y of life; sensitization (total and specific IgE; SPT reactivicity)	Yes	Yes[3]

							Atopic sensitisation – positive skin-prick test or positive RAST, or both			
**Kalliomaki 2003**						4 y				Yes[3b]
**Kalliomaki 2007**						7 y				Yes[3c]
**Kopp 2008**	Germany	pregnant women and their infants [≥1 family member (eg. mother, father, or sibling) with AD, AR or asthma and a confirmed allergic sensitization against an inhalant allergen]	5.0 x 10^9 CFU twice daily (n=54)	Microcrystalline cellulose (n=51)	for 4 – 6 w before delivery + 6 mo postnatally (3 mo BF women + 3 mo infants – added to water)	2 y	*AD* – pruritus, facial or extensor involvement, or both, and chronic relapsing course – which was fulfilled if the child had had eczema for >1 m0<br><br>*Recurrent Obstructive Bronchitis* – ≥ 5 episodes of wheezing bronchitis during the first 2 years	*the manifestation of AD at 2 y of life;<br><br>**egzema severity; Recurrent obstructive bronchitis	Yes	Yes[4]
**Ou 2012**	Taiwan	pregnant women and their infants [one family member (mother, father, or older sibling) with AD, AR, or asthma, and confirmed allergic sensitization against one or more of the common	1 x 10^{10} CFU (n=95)	Microcrystalline cellulose (n=96)	from 24 w gestation (second trimester) until delivery + 6 mo postnatally (BF women or non-breastfeeding neonates – added to water)	36 mo	*AD* – chronic or relapsing dermatitis with erythematous, scaly, or itchy rashes distributed on the face, neck, anterior chest wall, extensor areas or the flexural fold of extremities<br><br>*AR* – sneezing, running/blocked nose, or itchy eyes, in the absence of infection	* the point and cumulative prevalence of sensitization and developing of allergic diseases (AD, AR, asthma)<br><br>** improvement of maternal allergic symptom score and plasma immune parameters	Yes	No data

		allergen in the study region]					*Asthma* – recurrent, ≥3 episodes of wheezing/ coughing, requiring bronchodilator treatment<br><br>*Allergic sensitization* – positive specific IgE (> 0.7 kU/L) for one or more allergen			

[1] supported by Jack Brockhoff Foundation, the Murdoch Children's Research Institute, the Australian National Health and Medical Research Council and the Ilhan Food Allergy Foundation

[2] supported by the Clinical and Translational Science Institute (UL1 RR024131) at the University of California, San Francisco, and by the National Institutes of Health, HL 080074; Industry provided study products (eg. LGG and placebo capsules; pHF)

[3 a] supproted by the Finnish Foundation for Paediatric Research, the National Technology Agency of Finland, and the Allergy Research Foundation in southwest Finland; [3b] supported by the grants from the Academy of Finland and Turku University Hospital (EVO Fund); [3c] supported by the European Union (Early Nutrition Programming Project [EARNEST]), the Academy of Finland, and Turku University Hospital

[4] supported by the University of Freiburg and by Infectopharm (Heppenheim, Germany)

AD – atopic diseases; AR – atopic rhinitis; BF – breastfeeding; CBMC – cord blood mononuclear cells; DC – dendritic cell; Treg – lymphocytes T regulatory; RAST – radioallergosorbent assay; SPT – skin prick;

Table S2

Certainty assessment							№ of patients		Effect		Certainty
№ of studies	Study design	Risk of bias	Inconsistency	Indirectness	Imprecision	Other considerations	LGG	Control	Relative (95% CI)	Absolute (95% CI)	
Eczema - overall - LGG during pregnancy only - eczema 2 y											
1	randomized trials	not serious	not serious	not serious	not serious	none	42/122 (34.4%)	47/120 (39.2%)	**RR 0.88** (0.63 to 1.22)	**47 fewer per 1 000** (from 86 more to 145 fewer)	⊕⊕⊕⊕ HIGH
Eczema - overall - LGG to pregnant women & infants - eczema 18-24 mo											
3	randomized trials	not serious	not serious	serious [a]	not serious [a,b,c]	none	50/178 (28.1%)	56/174 (32.2%)	**RR 0.93** (0.49 to 1.76)	**23 fewer per 1 000** (from 164 fewer to 245 more)	⊕⊕⊕◯ MODERATE
Eczema - overall - LGG to pregnant women & infants - eczema 36-48 mo											
2	randomized trials	serious [b]	not serious	not serious	not serious	none	30/118 (25.4%)	41/118 (34.7%)	**RR 0.74** (0.43 to 1.26)	**90 fewer per 1 000** (from 90 more to 198 fewer)	⊕⊕⊕◯ MODERATE
Eczema - overall - LGG to pregnant women & infants - eczema 7 y											
1	randomised trials	serious [c]	not serious	not serious	not serious	none	23/53 (43.4%)	41/62 (66.1%)	**RR 0.66** (0.46 to 0.94)	**225 fewer per 1 000** (from 40 fewer to 357 fewer)	⊕⊕⊕◯ MODERATE
Eczema - overall - LGG to infants only -eczema 2 y											
1	randomized trials	not serious	not serious	not serious	not serious	none	26/92 (28.3%)	28/92 (30.4%)	**RR 0.93** (0.59 to 1.45)	**21 fewer per 1 000** (from 125 fewer to 137 more)	⊕⊕⊕⊕ HIGH

CI: Confidence interval; **RR:** Risk ratio

Explanations

a. High heterogeneity (I2 72%)

b. In one trial unclear randomization and allocation concealment. In one trial high attrition (>20%)

c. In one trial unclear blinding of participants and study personnel
